# A new species of *Erythrolamprus* from the oceanic island of Tobago (Squamata, Dipsadidae)

**DOI:** 10.3897/zookeys.817.30811

**Published:** 2019-01-15

**Authors:** John C. Murphy, Alvin L. Braswell, Stevland P. Charles, Renoir J. Auguste, Gilson A. Rivas, Amaël orzée, Richard M. Lehtinen, Michael J. Jowers

**Affiliations:** 1 Science and Education, Field Museum of Natural History, 1400 Lake Shore Drive, Chicago, IL 60616 USA; 2 North Carolina State Museum of Natural Sciences, 11 West Jones Street, Raleigh, NC USA; 3 Department of Biology, Howard University, 415 College Street NW, Washington, DC 20001 USA; 4 Department of Life Science, University of the West Indies, St. Augustine, Trinidad, WI; 5 Museo de Biologia, Facultad Experimental de Ciencias, Universidad del Zulia, Apartado Postal 526, Maracaibo 4011, Venezuela; 6 Division of EcoScience, Ewha Womans University, Seoul, 03760, Republic of Korea; 7 Interdisciplinary Program of EcoCreative, Department of Life Science, Ewha Womans University, Seoul, 03760, Republic of Korea; 8 Department of Biology, The College of Wooster, Wooster, OH USA 44691 USA; 9 CIBIO/InBIO (Centro de Investigação em Biodiversidade e Recursos Genéticos), Universidade do Porto, Campus Agrario De Vairão, 4485-661, Vairão, Portugal; 10 National Institute of Ecology, 1210, Geumgang-ro, Maseo-myeon, Seocheon-gun, Chungcheongnam-do, 33657, Republic of Korea

**Keywords:** cryptic species, evolutionary species concept, lowland montane rainforest, sky islands, systematics

## Abstract

Tobago is a small island on the southeast edge of the Caribbean Plate with a continental flora and fauna. Using DNA sequences from Genbank, new sequences, and morphological data from the snakes *Erythrolamprusepinephalus*, *E.melanotus*, *E.reginae*, and *E.zweifeli*, the species status of specimens of a Tobago snake previously considered to be *Erythrolamprusreginae* was assessed. *Erythrolampruszweifeli*, long considered a subspecies of *E.reginae*, was found to be a northern Venezuela-Trinidad endemic and the sister to *E.reginae*. The trans-Andean species *E.epinephalus* is shown to be non-monophyletic while the Costa Rican lineage of *E.epinephalus* is weakly supported as the sister to the Tobago population. The Tobago *Erythrolamprus* is described as a distinct taxon based upon five specimens from four localities in lower montane rainforest. Much of the new species range includes the Main Ridge Forest Reserve of Tobago, the oldest protected forest in the Western Hemisphere. All known locations fall within a 400-ha area, and its total geographic distribution is likely to be less than 4,566 ha. The restricted distribution of this new snake makes it a likely candidate for threatened status. The new species also becomes another biogeographic link between northern Venezuela and Tobago.

## Introduction

The Cordillera de Costa (CC) is a sky island archipelago that extends 925 km in an east-west orientation from western Venezuela, across the Northern Range of Trinidad to the island of Tobago. The CC is separated from the Andes by the Yaracuy River depression, and in the east, the CC is separated from the Guyana shield by the Llanos grasslands. The Gulf of Paria separates the Peninsula de Paria from Trinidad, and Trinidad is separated by 35 km of open water from Tobago. The CC formed between the late Cretaceous and the Miocene (Sisson et al. 2005). Rising and falling sea levels, marine incursions, changing climates, and tectonic events have continually remodeled the landscape isolating and reconnecting populations of organisms.

Tobago is at the eastern edge of the CC sky island complex and is slightly more than 300 km^2^, and its highest peak is about 576 m above sea level (ASL). The island has two physiographic regions: a flat coastal plain composed of a coral terrace in the southwest and the Main Ridge, a mass of metamorphic and igneous rocks, covered by dense tropical forest. The Main Ridge runs in a northeast-southwest direction.

Tobago’s snake fauna contains 23 species, and eleven of these belong to the Dipsadidae clade. Molecular studies on the Western Hemisphere snake clade Dipsadidae (or Dipsadinae) (Zaher et al. 2009; Vidal et al. 2010; Grazziotin et al. 2012) suggest *Erythrolamprus* Boie 1826 is not monophyletic unless most of the snakes formerly placed in the genera *Liophis* Wagler, 1830, *Leimadophis* Fitzinger, 1843, and *Umbrivaga* Roze, 1964 are included. This action increased the number of *Erythrolamprus* species from six, mostly coral snake mimics, to 50 species (Uetz and Jacob 2018) with a variety of color patterns and habits. Thus, *Erythrolamprus* became one of the most species-rich genera of Neotropical snakes. This arrangement has not been accepted by everyone (Wallach et al. 2014). Here we consider the genus *Erythrolamprus* in the broadest sense, including the species traditionally allocated to *Leimadophis*, *Liophis*, and *Umbrivaga*, acknowledging that future taxonomic changes are likely.

There is no known synapomorphy for the genus *Erythrolamprus* (Myers 2011). That said, members of the genus are usually less than 1.6 m in total length; nine scales are normally present on the crown; the number of dorsal scale rows is 15–19 and in some species they are reduced once, in others, they may be reduced twice posteriorly; apical pits may be present or absent on some or all of the scales; ventral counts range from 129–212; subcaudal counts range from 38–106; the temporal formula is usually 1+2; the preocular is usually single; the postoculars are usually two; upper labials are usually eight; lower labials are usually ten, and two pair of enlarged chin shields are present. *Erythrolamprus* ranges from Costa Rica southward to Argentina and occurs on both sides of the Andes as well as in the Lesser Antilles. Some taxa reach an elevation of 3,500 m ASL. Members of the genus have life styles that range from fossorial to terrestrial to semi-aquatic in habitats spanning rainforests, savannas, and páramo (Savage 2002).

### The genus *Erythrolamprus* in the Cordillera de la Costa

Eighteen species of *Erythrolamprus* occur in northern Venezuela, of these, two are Pantepui species: *E.trebbaui* (Roze 1958a), *E.ingeri* (Roze 1958b). The remaining 16 species are associated with the CC either as montane species, lowland species, or species that are not restricted by elevation. Eight of the 16 species occur on the Guyana Shield and seven species of *Erythrolamprus* are recognized on Trinidad and Tobago: *E.aesculapii* (Linnaeus, 1758); *E.bizona*Jan 1863; *E.cobellus* (Linnaeus, 1758); *E.melanotus* (Shaw, 1802), *E.ocellatus* Peters, 1868; *E.zweifeli* (Roze, 1959); and *E.reginae* (Linnaeus, 1758) (Murphy et al. 2018). Both *E.aesculapii* and *E.bizona* are coral snake mimics, and each is known from a single specimen from Trinidad (but better known from elsewhere in their ranges).

*Erythrolamprusocellatus* is a Tobago endemic, with a bright red dorsum and black ocelli, and is best considered an imperfect coral snake mimic, keeping in mind that there are no extant species of coral snakes on Tobago (Hodson and Lehtinen 2017). *Erythrolampruscobellus* is a semi-aquatic, lowland species; while *E.melanotus* and *E.zweifeli* are forest species often associated with stream-edge habitats and mountains from sea level to at least 2,000 m. However, in Venezuela, *E.zweifeli* is usually associated with montane environments.

Noting significant differences in coloration, as well as distinct ventral and subcaudal counts from *E.reginae*, Rivas et al. (2012) returned *Erythrolamprusreginaezweifeli* to species status. They noted *E.zweifeli* differs from *E.reginae* in having a salt-and-pepper dorsal pattern or a more uniform olive-green or olive-brown pattern. Wallach et al. (2014) concurred and recognized the elevation of *zweifeli* to species level. *E.reginae* has a dorsum with dense pale and dark paravertebral flecking. The two species also differ in subcaudal counts (69−88 in *E.zweifeli* as opposed to 55−78 in *E.reginae*) with the ranges overlapping, but different means. Following this arrangement, *E.zweifeli* occurs throughout the Cordillera de Mérida and the CC in Venezuela, including Trinidad.

### Natural history of Trinidad and Tobago´s *Erythrolamprus*

There are some ecological differences between the Trinidad and Tobago *Erythrolamprus.* The two poorly known coral snake mimics (*E.aesculapii*, *E.bizona*) are forest dwellers and snake predators (Campbell and Lamar 2004). Dietary differences between the two better known forest and forest-edge species are apparent. *Erythrolamprusmelanotus* feeds on the microteiid lizards in the genus *Bachia*, the rain frog *Pristimantisurichi*, the puddle frog *Engystomopspustulosus* and the gecko *Gonatodesvittatus*, and unidentified fish have been reported. *Erythrolampruszweifeli* feeds on stream frogs of the genus *Mannophryne*, hylid frogs, *Leptodactylusvalidus*, salamanders, lizards of the genus *Ameiva*, and small birds (Michaud and Dixon 1989; Murphy 1997; Esqueda et al. 2009). While the diets overlap the presence of *Bachia*, *Gonatodes*, and *Pristimantisurichi* in the diet of *E.melanotus* suggest it is hunting in more terrestrial situations in forests or at forest edges. *Mannophryne* in the diet of *E.zweifeli* suggests it is hunting along forested stream-edges. It supports the fact that *E.zweifeli* was the most common snake encountered during a study in a canal system used for water collection from a mountain stream located in Naiguatá, Venezuela (Silva et al. 1985; Silva and Valdez 1989).

*Erythrolamprusepinephalus* (Cope, 1862) is widespread and polytypic, ranging from Costa Rica to Ecuador, Colombia, and Venezuela and has not been previously associated with Trinidad or Tobago. The examination of a single specimen (USNM 22069) from Tobago led Dixon (1983b) to conclude that it was *Liophis* (= *Erythrolamprus*) *reginae* with an atypical color pattern that resembled a *Liophis* (= *Erythrolamprus*) *epinephalus* population from eastern Colombia. Dixon’s remark was the only mention of *E.epinephalus* associated with Trinidad and Tobago. He noted the most striking difference in the Tobago animal was a dorsolateral tan stripe not present in the Trinidadian *E.zweifeli*.

Here, we examine the genetic divergence and morphology of a Tobago snake, previously considered part of the *E.reginae* group, in an attempt to understand its phylogenetic relationship to other *Erythrolamprus* and the biogeography in northeastern South America.

## Materials and methods

Museum material examined (Appendix 1) included 105 specimens of five *Erythrolamprus* species. Snakes were examined for external morphological data; scale nomenclature follows Dixon (1983a, b). Museum acronyms are as follows:

**AMNH** American Museum of Natural History

**FMNH** Field Museum of Natural History

**EBRG** Museo de la Estación Biológica de Rancho Grande

**UMMZ** University of Michigan Museum of Zoology

**USNM** National Museum of Natural History

**UWIZM** University of the West Indies Zoology Museum

**MBLUZ** Museo de Biología, Universidad del Zulia

**MCNC** Museo de Ciencias Naturales, Caracas

Locality data was converted into coordinates using Google Earth. Measurements of the body and tail lengths were taken to the nearest millimeter; ventral scale count methods follow Dowling (1951). The anal plate and terminal scutes were not included in the number of ventrals or subcaudals. The dorsal scale row counts were made about ten ventrals behind the head, at mid-body, and about ten ventrals anterior to the vent. Values for paired head scales are given in left/right order. Scales were measured to the nearest 0.1 mm with the aid of a digital caliper and dissection microscope. Total length (TTL) and tail length (TL) measurements were taken to the nearest mm by carefully stretching the specimens along a ruler or placing a measuring tape along the length of the animal (Appendix 2). Statistical analyses were done with Excel-QI Macros (alpha = 0.05). Ventral and subcaudal counts were compared using ANOVA (Appendix 3).

DNA extraction, purification, and amplification protocols follow Jowers et al. (2013). Two mitochondrial gene fragments, 12S rDNA (primers 12Sa and 12Sb; Kocher et al. 1989), 16S rDNA (primers 16SL and 16SH; Palumbi 1996) and a nuclear gene fragment, c-mos (primers G73 and G74; Saint et al. 1998) were amplified. The lengths of the sequences were: 12S rDNA, 343 base pairs (bp); 16S rDNA, 425 bp; c-mos, 564 bp. We sequenced four *Erythrolamprusmelanotus* (GenBank accession numbers are shown in Appendix 4) from Trinidad (n = 1), Tobago (n = 3), two *E.zweifeli* from Trinidad, and a new undescribed *Erythrolamprus* sp. from Tobago. We downloaded all *Erythrolamprus* sequences for the same loci from Genbank and used *Xenodonhistricus* as the outgroup (Hodson and Lehtinen 2017).

**Figure 1.**
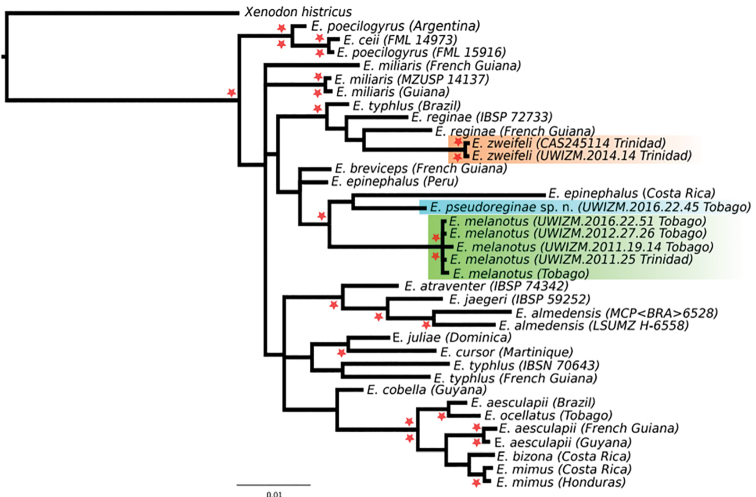
Bayesian inference tree of *Erythrolamprus* species from Genbank MtDNA 12S+16SrDNA+c-mos sequences (1332 bp). Red stars indicate Bayesian inference and ML posterior probabilities (> 95%) and bootstrap (> 70%) support values above and below nodes, respectively. Clade in orange shows *E.zweifeli*, in green *E.melanotus*, and in blue *E.pseudoreginae* sp. n. (AF158433) is from French Guiana, and *E.reginae* (JQ598983) is from Brazil.

Seaview v.4.2.11 (Gouy 2010) was used for preliminary alignments of sequences and were aligned thereafter in MAFFT (Katoh et al. 2002), and phylogenetic analyses were conducted using the concatenated mitochondrial and nuclear (12S+16S rDNA+c-mos) alignment (with a length of 1332 bp) using a partitioned model of substitution by gene fragment. The most appropriate substitution model for each gene partition was determined by the Bayesian Information Criterion (BIC) in PartitionFinder v.2 (Lanfear 2012). The best-fitting models for the ribosomal and c-mos fragments were as follows: 12S rDNA + 16S rDNA (TRN+I+G), c-mos first and second codon positions (TrNef+I) and c-mos third codon position (HKY). Phylogenetic relationships between taxa were inferred using the Bayesian Inference (BI) optimality criterion under the best fitting substitution model for each gene partition. MrBayes Huelsenbeck et al. (2001) was used with default priors and Markov chain settings, and with random starting trees. Each run consisted of four chains of 30 million generations, sampled every 1,000 generations. Runs were evaluated for convergence and mixing by observing and comparing traces of each parameter in Tracer v.1.6 (http://beast.bio.ed.ac.uk/tracer) (Rambaut et al. 2014). We considered effective sampling size (ESS) values > 200 to be good indicators of parameter mixing. Phylogenetic relationships (Figure 1) were also estimated using a Maximum Likelihood (ML) approach, as implemented in the software RAxML v7.0.4 (Silvestro and Michalak 2010), under the best partition scheme under the GTR model. All analyses were performed using the CIPRES platform (Miller et al. 2010). *P*-uncorrected distances were calculated in MEGA V7 (Kumar et al. 2016) under complete deletion of gaps and missing data.

### Molecular results

Runs showed high Effective Sample Size convergence (> 2300), indicating adequate sampling of the posterior distribution. The *p*-uncorrected distances between *L.epinephalus* from Costa Rica and *E.* sp. from Tobago were the highest of all terminal monophyletic clades (4.69%) indicating the high genetic divergence between both species (Appendix 5). The phylogenetic relationships of *Erythrolamprus* and the paraphyly of some species (*E.typhlus*, *E.poecilogyrus*, *E.epinephalus*, *E.aesculapii*) are similar to past published work (Hodson and Lehtinen 2017), suggesting the need for an in-depth systematic revision of the genus. Furthermore, the results show the paraphyly of *Erythrolamprusreginae*. *Erythrolamprusmelanotus* from Trinidad and Tobago are monophyletic, and the Trinidad specimen shows no genetic differentiation from the most common Tobago haplotype. *Erythrolamprus* sp. from Tobago is the sister clade to *E.epinephalus* from Costa Rica. This clade, composed by the three species (*E.melanotus* + *E.epinephalus*+*E.* sp. Tobago), is strongly supported in the Bayesian analyses. The Trinidadian *E.zweifeli* form the sister clade to *E.reginae* from Guyana but are a distinct lineage.

### Morphological results

**Figure 2.**
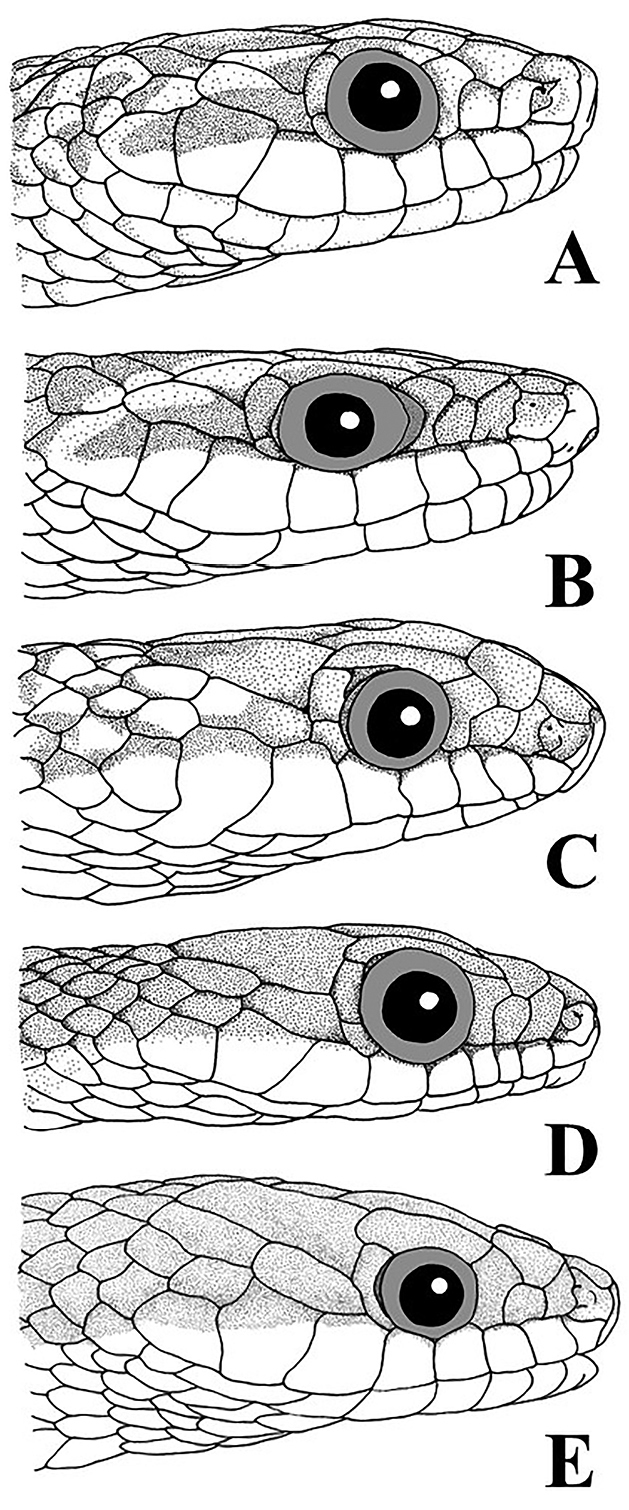
A comparison of the five members of the *Erythrolamprusreginae* group. **A***E.reginae* for Guyana (FMNH 30959) **B***E.zweifeli* from Venezuela (FMNH 204477) **C***E.melanotus* from Tobago (UWIZM.2012.42.19) **D***E.pseudoreginae* sp. n. from Tobago (FLMNH 91621) **E***E.epinephalus* from Venezuela (MBLUZ 1502).

**Figure 3.**
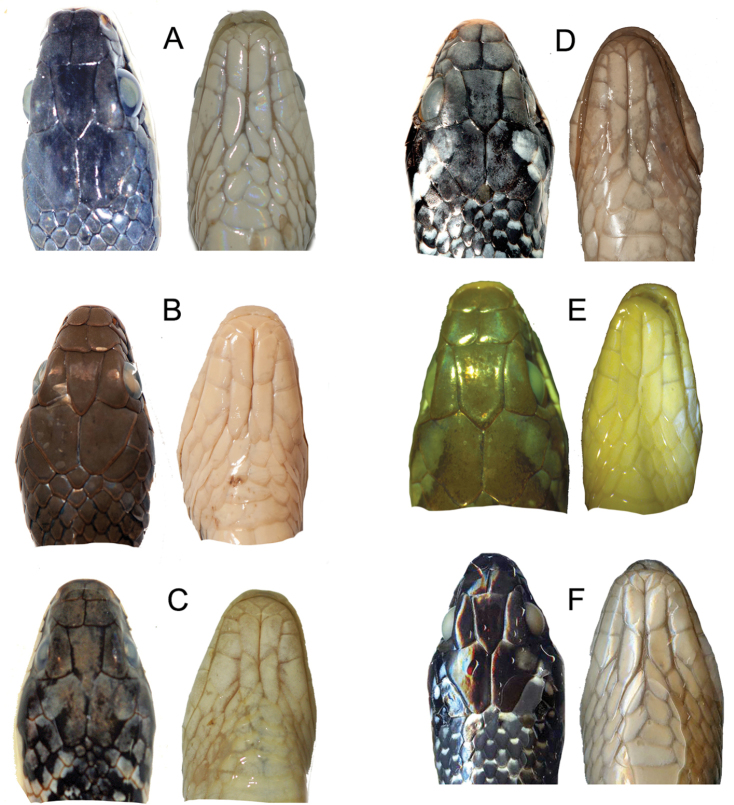
A comparison of the scale arrangements on the crowns and ventral heads of the *Erythrolamprus* taxa under discussion. **A***E.pseudoreginae* sp. n. from Tobago **B***E.epinephalus* from Venezuela MBLUZ 1501 (dorsal view) and 1500 (ventral view) **C, D** Salt and pepper morph of *E.zweifeli* from Trinidad and Venezuela **E** An olive-brown morph of *E.zweifeli* Trinidad, FMNH 215827 **F** A melanistic morph of *E.zweifeli* from Venezuela EBRG 2745.

**Figure 4.**
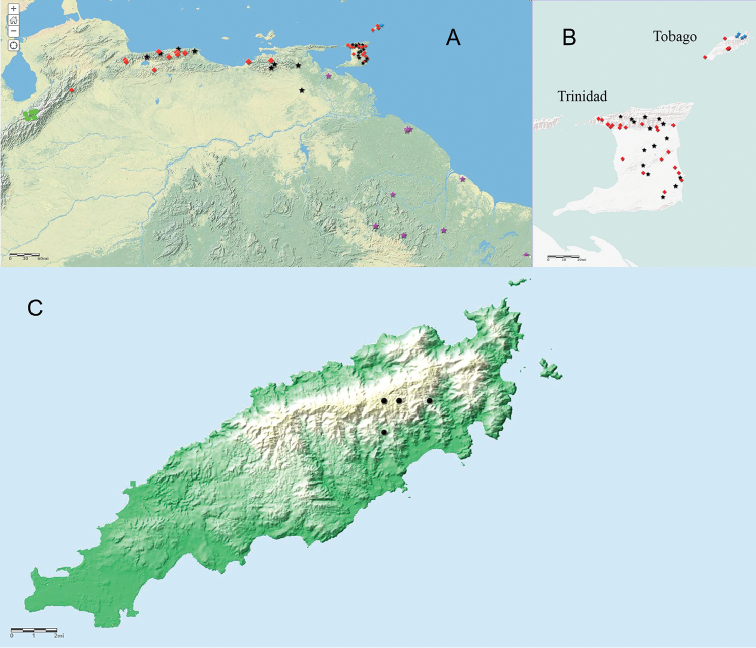
Geographic distribution of the five species of *Erythrolamprus* under discussion in this paper. **A&nbsp**;The distribution of the species of *Erythrolamprus* under discussion in northern Venezuela and Trinidad and Tobago **B** More detailed view of the distribution on Trinidad and Tobago **C** Tobago with the known localities for *E.pseudoreginae* sp. n. Note that two of the markers closely overlap. Key: black stars = *E.zweifeli* from Cordillera de Costa in Venezuela and the island of Trinidad; green circles = *E.epinephalus* from the Cordillera de Mérida, Venezuela. Note that these markers denote the closest population to Tobago based on Roze (1966). Specimens examined came from several different locations. Purple stars = *E.reginae* from the Guianas including Orinoco Delta in Venezuela; red stars = *E.melanotus* from Venezuela, Trinidad, and Tobago; blue star = *Erythrolampruspseudoreginae* sp. n. on Tobago.

Figure 2 shows the similarities in the architecture of the scales when *Erythrolamprus* are viewed in profile. They all have a single preocular, two postoculars, and eight upper labials; the second and third upper labials are in contact with the loreal, the fourth and fifth border the orbit, and the temporal formula is 1+2. Figure 3 compares the crowns and chins of four of these species (including *E.zweifeli* from three different populations). They all share nine plate-like scales on the crown in similar proportions and two pair of enlarged chin-shields. Figure 4 illustrates the distribution of the five species in northern South America, Trinidad, and Tobago.

Comparisons and summaries of the meristic characters for taxa under consideration are given in Table 1. Ventral counts for all *Erythrolamprus* taxa under consideration have ranges that overlap, although they have different means, some of which are significantly different. The ranges for the subcaudal counts are similar. The Tobago *E.pseudoreginae* sp. n. can be separated from *E.melanotus* but not the other taxa. The results of single factor ANOVAs are presented in Appendix 2. Some support the separation of *E.zweifeli* from *E.reginae*, *E.zweifeli* from the Tobago *E.pseudoreginae* sp. n., and *E.reginae* from the Tobago *E.pseudoreginae* sp. n.

**Table 1.** A comparison of the meristic and color pattern data for the five taxa in *Erythrolamprus* in the Trinidad and Tobago area. Key: * based on our counts for Venezuelan specimens.

	*** E. melanotus ***	*** E. reginae ***	*** E. zweifeli ***	***E.pseudoreginae* sp. n.**	*** E. epinephalus ***
Number of specimens	12	14	44	5	6
stripe on rows	4–5	3–4	3–4	3–4–5	variable
ventral range	139–154	129–147	134–157	143–154	144–157*
mean ventrals ± SD	146.66 ± 4.36	138.35 ± 4.71	142.54 ± 3.98	147.5 ± 3.35	151.33 ± 3.38
subcaudal range	53–58	68–79	72–85	76–79	65–75*
mean subcaudals ± SD	55.2 ± 1.4	72.0 ± 7.14	79.9 ± 4.20	77.5 ± 1.5	68.2 ± 3.38
postocular stripe	present	indistinct	present	indistinct	variable
ventral color	yellow	yellow to pale orange, usually with black checks	red with black checks, some ventrals solid black	uniform yellow to red with scattered fine speckling	variable
apical pit present	yes	yes	no	yes	yes

**Figure 5.**
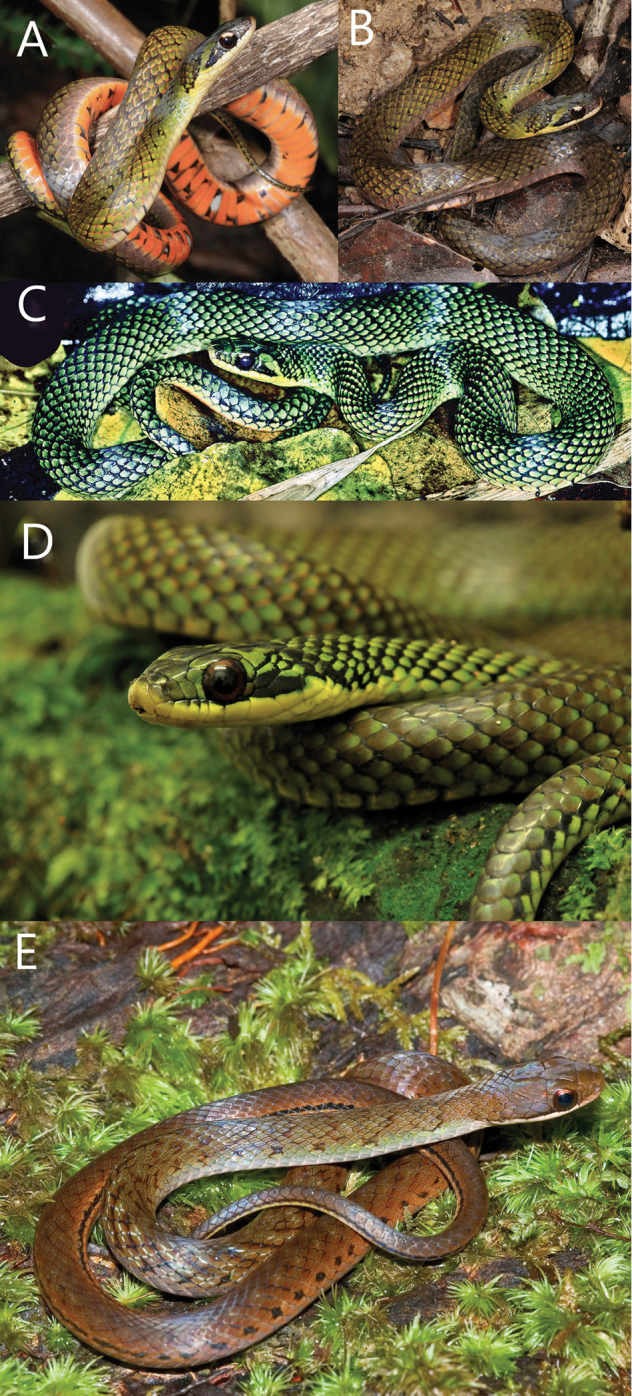
**A, B** Variations in the olive color morph of *Erythrolampruszweifeli* from Trinidad (photographs by Michael Patrikeev) **C** the middle photo shows the “salt and pepper” morph that occurs at higher elevation (photograph by JCM). Both color morphs are included in our molecular sample **D***E.zweifeli* Rancho Grande, Parque Nacional Henri Pittier, Luis A. Rodriguez J. **E** the Royal Snake, *Erythrolamprusreginae* from Kaiteur, Guyana (photograph by P Kok).

**Figure 6.**
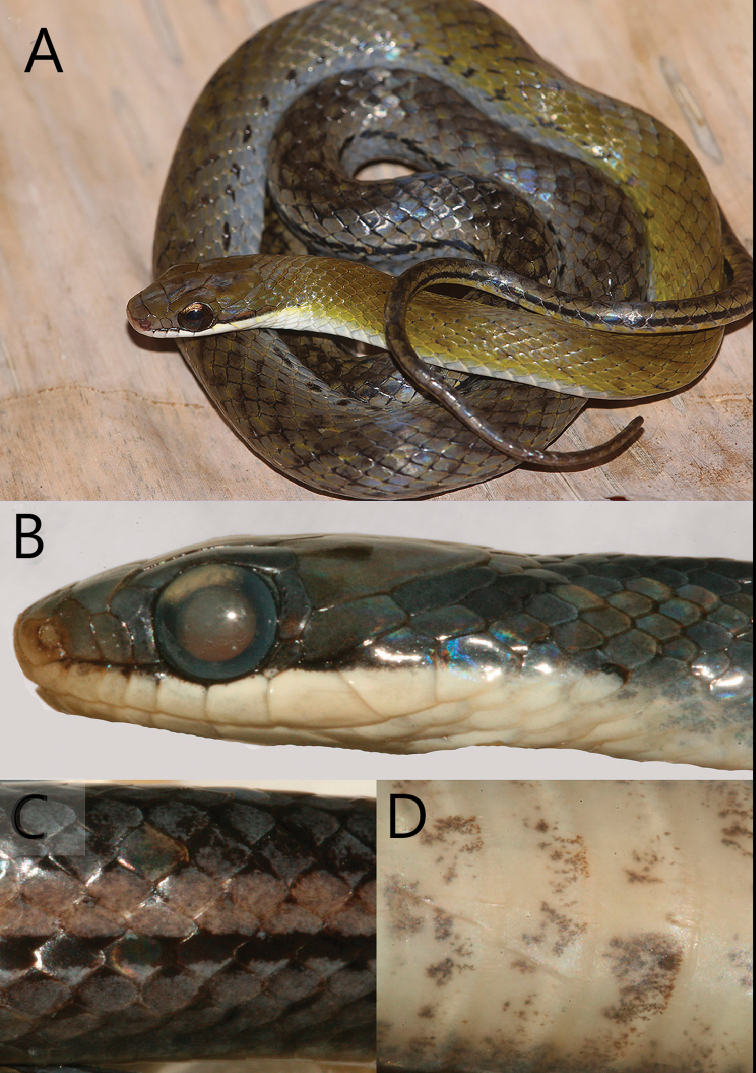
*Erythrolampruspseudoreginae.***A**UWIZM 2016.22.45, holotype **B–D** FLMNH 91621 from Gilpin Trace, on Tobago’s Main Ridge. **B** Profile. Of the four specimens examined this was the only one that had nine upper labials (on one side only) **C** The posterior lateral stripe bordered by a dorsal light stripe **D** Venter mostly uniform with patches of scattered pigment.

Substantial genetic differences (0.047) (Appendix 5) and relatively minor morphological differences (different means for ventral counts, distinctive coloration, the absence of apical pits on dorsal scales) and its geographic isolation support the description of the Tobago population as a new species. Figure 5 compares the color morphs of *Erythrolampruszweifeli* found in Trinidad and Venezuela with *Erythrolamprusreginae* from Guyana. Figure 6 illustrates *E.pseudoreginae* sp. n.

#### Erythrolamprus pseudoreginae sp. n.

Taxon classificationAnimaliaSquamataDipsadidae

http://zoobank.org/B5FAE467-C240-4EBB-9DA5-B3D44998757E

Figures 2D , 3A , 6

Liophis sp. Hardy 1982: 86.
Liophis reginae [ssp.] Dixon 1983b: 12.

##### Material.

**Holotype.**UWIZM.2016.22.45 collected 13 June 2016 by Alvin L. Braswell and Renoir J. Auguste on Gilpin Trace Trail, 8.5 km NNW Roxborough, St. John, Tobago (~11°16'55"N; 60°37'12"W, about 493 m ASL) at 0900 hrs. **Paratypes.** TOBAGO: St John: FLMNH 91621 Gilpin Trace Trail, 5.3 mi NNW Roxborough, 11°16'N, 60°37'W collected on 17 July by Kurt Auffenberg. USNM 228069 south of Charlotteville, at first creek crossing on Pigeon Peak Trace 11°17'N, 60°36'W collected 12 May 1979 at (14:00 hrs) by Dave Stephens; USNM 325089 NW of Roxborough on Gilpin Trace, ca. 0.5 mi from its junction with Roxborough-Bloody Bay Road, collected 11 November 1992; USNM 539191 approx. 6 km (airline) NNW of Roxborough, 0.5 mi from upper entrance of Gilpin Trace and Roxborough - Parlatuvier Road, 11°17'N, 60°35'W collected 11 July 2000.

##### Diagnosis.

Ventrals 143–154; subcaudals 76–79; second pair of chin shields longest; some anterior dorsal scales have an apical pit; lateral stripe on scale rows 3–4–5, dark stripe (row 3) and a pale stripe (rows 4–5) on posterior body and tail, the black stripe continues to the forebody as a series of black spots on scale row three; and the ventral surface has scattered flecks of pigment toward mid-body. Otherwise, the belly is uniform cream with fine speckling in preserved material, and red in life, tail uniform cream in preservative, red in life.

##### Description of the holotype.

UWIZM.2016.22.45, an adult male, 525 mm total length, 148 mm tail; tail 28% of SVL. Rostral barely visible from above, broader than tall; internasals paired, shorter than prefrontal; frontal pentagonal; parietals longer than frontal; four post parietals; nasal divided, first lobe does contact the second labial; loreal subrectangular, higher than long, contacts upper labials 2–3; preocular single, T-shaped, contacts upper labials 3–4; postoculars 2/2, upper largest; temporals 1+2, primary temporal contacts upper labials 6–7/6–7; upper labials 8/8; 4–5 in orbit; lower labials 4/5 contact anterior chin shields, total of nine in contact with both pairs; lower labials 9/10; three gular scales; dorsal scales are smooth, some have a single apical pit, they are in 17 rows at mid body and reduced to 15 rows anterior to the cloaca; 146 ventrals; 77 subcaudals.

In life the crown is dark moss green with black spots, the upper labials are cream, with a dark stripe on the upper edge that runs from nasal to orbit, and widens posteriorly onto the temporals. Dorsal spots on scale rows 2–3 about two ventrals apart, start above the 12^th^ ventral, and coalesce into a stripe at about the 96^th^ ventral and extend posteriorly to the tip of the tail; lateral stripe mostly on scale row three on body, goes onto scale row one on tail. About one-third down the body, about ventral 40, scale rows 1−4 blue-gray, row five is brown, row six and above blue-gray; except for the mossy green on the anteriormost dorsal surface for about 40 ventrals. Ventral surface mostly uniform yellow to orange with light mottling starting about the 50^th^ ventral; tail has a mid-line zigzag stripe.

**Variation**: The smallest specimen measured 347 mm SVL with a 129 mm tail; the largest specimen 420 mm SVL with a 119 mm damaged tail. Dorsal scale rows 17–17–15. Ventrals range from 143–154 (n = 5, X = 147.5, SD = 3.35); subcaudals 76–79 (n = 2, X = 77.5, SD = 1.5). Upper labials eight or nine, 2–3 contact loreal, 4–5 border the orbit (one specimen has 5–6 bordering the orbit on one side), the tallest can be seventh, (or eight if nine labials are present); the sixth labial is the largest in the area. Loreal is quadrangular to pentagonal. Lower labials 9–10; first four or five contact the anterior chin shields. Longest pair of chin shields is the second. Eye diameter is greater than eye-nostril distance. The dark posterior lateral stripe is usually on scale rows 2–3–4, but one specimen has it on scale rows 2–3 only.

Color in life. The following is based on the holotype (Figure 6) and a color photograph in Brown (2013). Crown and face olive brown, upper labials white, a short black subocular stripe extends from the nasal scale under the eye and posteriorly to the last labial. Immediately behind the head, the interstitial skin is yellow; dorsum brown with an indistinct vertebral stripe and scales partially edged with black pigment most obvious on posterior two-thirds of the body. First three scale rows are blue-gray and separated from brown dorsum by a row of black spots.

Color in alcohol. Head, body, and tail dark blue to brown with a black stripe on the posterior lateral body that becomes a series of dark spots extending anteriorly on the body. The belly is a uniform cream with fine speckling of pigment.

##### Comparisons.

*Erythrolampruspseudoreginae* sp. n. differs from *E.zweifeli* in the presence of apical pits on some dorsal scales, an almost uniform yellow to red venter, and a dark stripe on the posterior body on scale rows 3–4 bordered above by a pale stripe on scale row five. The new species lacks the well-defined postocular stripe that runs from the postocular scales across the temporals to a point just above the rictus in most *E.zweifeli*. In *E.zweifeli* the postocular stripe may also have a pale dorsal border.

*Erythrolampruspseudoreginae* sp. n. differs from all populations of *E.epinephalus* in having more than 75 subcaudal scales, except for some Venezuelan and Colombian populations. The *E.epinephalus* populations with more than 75 subcaudals have a dorsal or ventral pattern that includes transverse bars, black checks, or a pattern with irregular black spots on the outer edges of the ventral scales that may extend onto the first row of dorsal scales (Dixon 1983a, Escalona 2017).

The new species differs from *Erythrolamprusreginae* in having a uniform venter (*E.reginae*) has yellow to orange venter with black checks, and a dark stripe on the last fourth of the body on scale rows 3–4 which is not bordered by a pale stripe. *Erythrolampruspseudoreginae* sp. n. has uniform yellow to red ventral surface and a very distinctive, pale posterior lateral stripe on row five above the black stripe on rows 3–4 that extends anteriorly as a row of dark spots. *Erythrolamprusreginae* has fewer ventrals and a lower mean ventral count than *E.pseudoreginae* sp. n.

The pattern will readily distinguish it from the two coral snake mimics (*Erythrolamprusaesculapii* and *E.bizona*) which are on Trinidad but not Tobago. The endemic Tobago Red Snake, *E.ocellatus*, has a bright red dorsum with black ocelli. The semi-aquatic *Erythrolampruscobellus* has a uniform dark green or black dorsum and is known from Trinidad but not Tobago. The absence of a black stripe five scale rows wide on the vertebral line separates it from Shaw’s Black Back Snake, *E.melanotus*, a species known from both islands.

##### Distribution.

It occurs in northeastern Tobago and appears to be restricted to the forested ravines along the crest of the Main Ridge (Fig. 4). Tobago’s Main Ridge is about 16 km long and covered with lower montane rain forest on schist soil above 224 m ASL. The ridge crest reaches elevations of 487–576 m ASL and forms steep terrain with deep gullies and fast-moving streams. The area receives about 318 cm of rainfall per year, and no month receives less than 10 cm (Beard, 1944). Tobago’s Main Ridge Forest Reserve is the oldest protected forest in the Western Hemisphere (since April 1776) and encompasses 3958 hectares. At this writing, five specimens of *Erythrolampruspseudoreginae* sp. n. are known, all of which came from the northeast end of the Main Ridge. The locality and elevation data available suggest it occurs within an area of about 400 ha at elevations between 430–500 m ASL. Three types of rainforest occur on Tobago: lowland rainforest covers 4,844 ha, lower montane rainforest covers 4,566 ha, and xerophytic rainforest covers 937 ha (Helmer et al. 2012). All of the localities for *E.pseudoreginae* sp. n. fall within the lower montane rainforest, suggesting its maximum area of occupancy may be 4,566 ha, if it is restricted to that forest type.

##### Natural History.

*Erythrolampruspseudoreginae* sp. n. is diurnal, all of the specimens with time of collection data were found in the morning or afternoon. Nothing is known about the diet and reproduction of this snake. Its close relatives have been reported to eat anurans, and it likely preys upon small ground-dwelling frogs.

##### Conservation.

Given the restricted distribution of this snake as well as the fact that most, if not all, of its distribution lies within the oldest protected forests in the Western Hemisphere it may be assumed that it is well protected. However, as the climate changes the microclimate found in the lowland montane rainforest may be expected to change and potentially make the local environment inhospitable for this species and the other endemic taxa found here.

##### Etymology.

The epithet *pseudoreginae* was chosen because prior investigators considered this snake to be *Liophisreginae*. We suggest Tobago Stream Snake as the common English name for this snake.

## Discussion

*Erythrolampruspseudoreginae* becomes the fifty-first species in the genus, and the eleventh member of the Tobago herpetofauna closely associated with the Main Ridge. The list of Main Ridge species includes the frogs *Mannophryneolmonae*, *Pristimantischarlottevillensis*, *P.turpinorum*, *Hyalinobatrachiumorientale*; the lizards Bachiacf.flavescens, *Gonatodesocellatus*, Anoliscf.tigrinus; and the snakes *Atractusfuliginosus*, *Erythrolamprusocellatus*, and *Leptophishaileyi*.

Most of the Main Ridge endemic species seem to have their closest living relatives in the Costal Ranges of Venezuela as opposed to the more proximal island of Trinidad or the Guiana Shield. The Coastal Range endemic *Mannophryneriveroi* is the sister to *M.olmonae* (Manzanilla et al. 2009, Lehtinen et al. 2011). Tobago’s *Pristimantischarlottevillensis* appears to be most closely related to *P.terraebolivaris* and members of the *Pristimantisconspicillatus* group (Hedges et al. 2008). Jowers et al. (2015) proposed a Pliocene land bridge connection between Tobago and Venezuela to explain the presence of *Hyalinobatrachiumorientale* on Tobago and northeast Venezuela. An alternative explanation is that Tobago was accreted to Venezuela on its movement to its current position.

With this study, only 21 of the 51 named *Erythrolamprus* species have been included in molecular studies; thus, the tree contains only 41% of the known species in the genus. Therefore, its topography is likely to change with additional taxa from more locations. *Erythrolamprusreginae* and *E.epinephalus* are polytypic and given their distributions and morphological variation they represent a considerable challenge to resolving the lineages found within these taxa. Some of the color patterns have evolved multiple times in the different lineages and when combined with the conserved morphology, separating these taxa by morphology becomes a conundrum. It seems likely that some of the currently recognized subspecies will be found more closely related to lineages other than the one they are currently assigned.

The phylogenetic analyses suggest part of *E.reginae* is the sister to *E.zweifeli*. The results show for the first time the Trinidadian *E.melanotus*, has no genetic divergence from the most common haplotype from Tobago. This lack of differentiation suggests some recent or ongoing gene flow between islands. The position of *E.ocellatus* from Tobago suggests that it forms a highly divergent lineage to the remaining Tobago´s *Erythrolamprus* and may indicate a different time of colonization.

## Supplementary Material

XML Treatment for Erythrolamprus pseudoreginae

## Appendix 1

**Table:** Locality data for specimens examined in this study. Coordinates represent georeferencing from Google Earth, variation from the exact collecting locality is expected.

Species	Voucher	Country	Coordinates
* epinephalus *	MBLUZ 1500	Venezuela	10°19'N; 72°35'W
	MBLUZ 1501	Venezuela	10°19'N; 72°35'W
	MBLUZ 1502	Venezuela	10°19'N; 72°35'W
	MBLUZ 1503	Venezuela	10°19'N; 72°35'W
	MCNC 5677	Venezuela	07°39'N; 72°21'W
	MCNC 7875	Venezuela	07°29'N; 72°27'W
* melanotus *	FMNH 61669	Colombia	07°09'N; 75°21'W
	FMNH 61670	Colombia	no specific locality
	FMNH 121224	Colombia	04°09'N; 73°38'W
	FMNH 165341	Colombia	10°26'N; 75°22'W
	FMNH 165399-408	Colombia	10°26'N; 75°22'W
	FMNH 165498	Colombia	10°26'N; 75°22'W
	FMNH 165644	Colombia	10°26'N; 75°22'W
	FMNH 165645	Colombia	10°26'N; 75°22'W
	FMNH 217232	Trinidad	10°43'N; 61°17'W
	FMNH 218779	Trinidad	10°43'N; 61°17'W
	FMNH 49947-50	Trinidad	10°29'N; 61°28'W
	FMNH 49945-46	Trinidad	10°16'N; 61°1'W
	FMNH 5674	Trinidad	10°39'N; 61°30'W
	FMNH 77902-03	Trinidad	10°39'N; 61°30'W
	FMNH 190749	Trinidad	10°09'N; 61°30'W
	FMNH 49938-44	Trinidad	10°34'N; 61°15'W
	FMNH 69778	Venezuela	10°28'N; 67°07'W
* reginae *	AMNH 3595	“Guiana”	no specific locality
	USNM 164210	Guyana	8°12'N; 59°46'W
	USNM 164208	Guyana	8°12'N; 59°46'W
	FMNH 30959	Guyana	10°29'N; 61°28'W
	FMNH 30962	Guyana	no specific locality
	UMMZ 53901	Guyana	no specific locality
	UMMZ53912	Guyana	no specific locality
	UMMZ 53968	Guyana	no specific locality
	UMMZ 53969	Guyana	no specific locality
	AMNH 17680	Guyana	6°47'N; 58°09'W
	FMNH 56149	Peru	4°36'S; 74°10'W
	FMNH 40234	Peru	11°48'S; 70°48'W
	AMNH 8132	Suriname	5°51'N; 55°12'W
	AMNH 4436	Venez. or Brazil	no specific locality
* pseudoreginae *	USNM 539191	Tobago	11°17'N; 60°35'W
	UWIZM.2016.22.45	Tobago	11°17'N; 60°36'W
	UWIZM 91621	Tobago	11°16'N; 60°37'W
	USNM 325089	Tobago	11°17'N; 60°36'W
	USNM 228069	Tobago	11°17'N; 60°36'W
*Erythrolamprus* sp.	USNM 549328	Guyana	05°17'N; 60°45'W
* zweifeli *	FMNH 215827	Trinidad	10°43'N; 61°17'W
	FMNH 217226-27	Trinidad	10°43'N; 61°17'W
	FMNH 219615	Trinidad	10°43'N; 61°17'W
	USNM 17757-58	Trinidad	10°43'N; 61°17'W
	FMNH 49957-58	Trinidad	10°28'N; 61°28'W
	UWIMZ 2010.12.110	Trinidad	10°43'N; 61°25'W
	UWIMZ 2010.12.201	Trinidad	10°45'N; 61°26'W
	UWIMZ 2010.12.108a, b	Trinidad	10°16'N; 61°1'W
	UWIMZ 2010.12.107	Trinidad	no specific locality
	UWIMZ 2010.12.109	Trinidad	no specific locality
	USNM 252682-83	Trinidad	10°45'N; 61°17'W
	USNM 286922	Trinidad	10°30'N; 61°16'W
	AMNH 137503	Venezuela	10°01'N; 67°17'W
	AMNH 98260	Venezuela	10°06'N; 63°06'W
	USNM 217197	Venezuela	02°37'N; 66°19'W
	FMNH 120986	Venezuela	10°01'N; 67°17'W
	FMNH 204477	Venezuela	10°01'N; 67°17'W
	UMMZ 124225	Venezuela	10°01'N; 67°17'W
	UMMZ 124227-33	Venezuela	10°01'N; 67°17'W
	UMMZ 128390	Venezuela	10°01'N; 67°17'W
	USNM 217198	Venezuela	10°15'N; 68°21'W
	USNM 196332	Venezuela	10°13'N; 66°25'W
	AMNH 67877	Venezuela	10°06'N; 63°06'W
	AMNH 29317	Venezuela	10°09'N; 64°17'W
	AMNH 29332	Venezuela	10°29'N; 66°07'W
	AMNH 29317	Venezuela	10°22'N; 63°17'W
	FMNH 17833-36	Venezuela	10°22'N; 63°17'W
	AMNH 29332	Venezuela	10°09'N; 64°17'W

## Appendix 2

**Table:** Morphometric data and sex for specimens of *Erythrolamprus* species examined. Key: m = male, f = female; j = juvenile; SVL = snout vent length mm; tail mm; D1–3 dorsal scale rows at anterior, midbody, and posterior body); V = ventral scales; S = subcaudal scales; nd = no data.

Museum	Voucher	Species	Sex	svl	tail	D1	D2	D3	V	S
MBLUZ	1500	epinephalus	?	335	88 d	17	17	15	153	51+
MBLUZ	1501	epinephalus	?	330	112	17	17	15	155	75
MBLUZ	1502	epinephalus	?	280	95	17	17	15	153	69
MBLUZ	1503	epinephalus	f	340	19	17	17	15	157	67
MCNC	5677	epinephalus	m	355	112	17	17	15	144	65
MCNC	7875	epinephalus	m	345	120	17	17	15	146	65
FMNH	165402	* melanotus *	f	332	90	17	17	15	142	54
FMNH	49946	* melanotus *	f	291	61	17	17	15	144	57
FMNH	49947	* melanotus *	f	230	52	17	17	15	139	53
FMNH	49950	* melanotus *	f	307	77	17	17	15	nd	nd
FMNH	190749	* melanotus *	f	305	72	17	17	15	142	54
FMNH	165644	* melanotus *	m	358	92	17	17	15	149	55
FMNH	165498	* melanotus *	m	325	97	17	17	15	144	54
FMNH	165407	* melanotus *	m	350	85	17	17	15	147	55
FMNH	49949	* melanotus *	m	271	76	17	17	15	147	56
FMNH	77903	* melanotus *	m	275	76	17	16	15	152	58
FMNH	69778	* melanotus *	m	370	72+	17	17	15	154	nd
FMNH	121224	* melanotus *	m	282	81	17	16	15	149	57
FMNH	61670	* melanotus *	nd	310	81	17	17	15	151	55
AMNH	4436	* reginae *	f	355	128	17	17	15	144	74
UMMZ	53912	* reginae *	f	420	117	17	17	15	133	79
UMMZ	53969	* reginae *	f	415	nd	17	17	15	136	nd
USNM	164210	* reginae *	f	428	nd	17	17	15	139	nd
AMNH	3595	* reginae *	f	443	nd	17	17	15	136	nd
FMNH	40234	* reginae *	j	128	44	17	17	15	147	74
AMNH	17680	* reginae *	m	313	120	17	17	15	137	73
AMNH	8132	* reginae *	m	445	nd	17	17	15	142	nd
FMNH	30959	* reginae *	m	443	d	17	17	15	139	nd
FMNH	56149	* reginae *	m	419	117	17	17	15	145	55
UMMZ	53901	* reginae *	m	428	186	17	17	15	139	78
UMMZ	53968	* reginae *	m	474	210	17	17	15	135	75
USNM	164208	* reginae *	m	308	nd	17	17	15	136	nd
FMNH	30962	* reginae *	nd	nd	nd	17	17	15	129	68
USNM	539191	* pseudoreginae *	f	408	nd	17	17	15	148	nd
USNM	228069	* pseudoreginae *	f	347	129	17	17	15	143	76
USNM	539191	* pseudoreginae *	f	408	nd	17	17	15	148	nd
FLMNH	91621	* pseudoreginae *	m	420	119	17	17	15	146	nd
FLMNH	91621	* pseudoreginae *	m	420	119	17	17	15	146	nd
USNM	325089	* pseudoreginae *	m	408	158	17	17	15	154	79
USNM	549328	*Erythrolamprus* sp.	m	361	117	17	17	15	148	64
AMNH	137503	* zweifeli *	f	456	167	17	17	15	146	83
FMNH	17836	* zweifeli *	f	380	165	17	17	15	138	82
FMNH	204477	* zweifeli *	f	454	180	17	17	15	142	85
UMMZ	128390	* zweifeli *	f	nd	nd	17	17	15	141	nd
UMMZ	124232	* zweifeli *	f	375	144	17	17	15	144	nd
UMMZ	1288390	* zweifeli *	f	402	162	17	17	15	141	84
USNM	17757	* zweifeli *	f	471	187	17	17	15	143	76
USNM	252683	* zweifeli *	f	236	nd	17	17	15	140	nd
USNM	217197	* zweifeli *	f	434	167	17	17	15	134	72
USNM	252683	* zweifeli *	f	236	nd	17	17	15	140	nd
UWIZM	2010.12.109	* zweifeli *	f	245	nd	17	17	15	nd	nd
UWIZM	2010.12.107	* zweifeli *	f	355	152	17	17	15	139	79
UWIZM	2010.12.201	* zweifeli *	f	401	158	17	17	15	143	79
UWIZM	2010.12.109	* zweifeli *	f	245	nd	17	17	15	nd	nd
FMNH	17833	* zweifeli *	j	172	63	17	16	15	140	80
FMNH	17835	* zweifeli *	j	152	56	17	17	15	144	85
UMMZ	124229	* zweifeli *	j	305	117	17	17	15	139	83
UMMZ	124230	* zweifeli *	j	184	67	17	17	15	141	80
UMMZ	124227	* zweifeli *	j	nd	nd	17	17	15	142	84
UMMZ	124231	* zweifeli *	j	185	62	17	17	15	145	nd
UWIZM	2010.12.108b	* zweifeli *	j	136	45	17	17	15	134	75
AMNH	29317	* zweifeli *	m	365	152	17	17	15	145	79
AMNH	29332	* zweifeli *	m	297	114	17	17	15	151	76
AMNH	R-29317	* zweifeli *	m	369	148	17	17	15	143	82
AMNH	29332	* zweifeli *	m	322	115	17	17	15	142	74
AMNH	67877	* zweifeli *	m	361	148	17	17	15	149	82
FMNH	17834	* zweifeli *	m	384	101+	17	15	15	141	nd
FMNH	217226	* zweifeli *	m	340	139	17	17	15	138	77
FMNH	219615	* zweifeli *	m	d	nd	17	17	15	142	nd
FMNH	49957	* zweifeli *	m	398	174	17	15	15	145	79
FMNH	49958	* zweifeli *	m	456	nd	17	17	15	145	nd
FMNH	215827	* zweifeli *	m	354	155	17	16	15	140	78
FMNH	217227	* zweifeli *	m	367	157	17	15	15	141	nd
FMNH	120986	* zweifeli *	m	386	143	17	17	15	142	83
FMNH	215827	* zweifeli *	m	354	155	17	16	15	140	78
FMNH	217227	* zweifeli *	m	367	157	17	15	15	141	nd
UMMZ	124233	* zweifeli *	m	394	163	17	17	15	142	83
UMMZ	124225	* zweifeli *	m	363	149	17	17	15	144	80
UMMZ	124228	* zweifeli *	m	415	135+	17	17	15	143	nd
USNM	17758	* zweifeli *	m	349	nd	17	17	15	142	nd
USNM	252682	* zweifeli *	m	370	160	17	17	15	141	83
USNM	286922	* zweifeli *	m	165	55	17	17	15	149	80
USNM	196332	* zweifeli *	m	430	170	17	17	15	145	78
USNM	217198	* zweifeli *	m	509	203	17	17	15	140	75
USNM	252682	* zweifeli *	m	370	160	17	17	15	141	83
USNM	286922	* zweifeli *	m	165	55	17	17	15	149	80
AMNH	98260	* zweifeli *	nd	492	nd	17	16	15	144	nd
UWIZM	2010.12.108a	* zweifeli *	nd	360	nd	17	17	15	146	nd
UWIZM	2010.12.110	* zweifeli *	nd	373	157	17	15	15	138	80

## Appendix 3

**Table:** (**A**) compares the single factor ANOVA results for ventral counts and (**B**) compares the single factor ANOVA results for subcaudal counts. Statistically significant results that resulted in the rejection of the null hypothesis are in bold.

A. ventrals		
	* zweifeli *	* pseudoreginae *
		**p = 0.003**
* zweifeli *		**df = 53**
	**p = 0.00**	**p = 0.00**
* reginae *	**df = 59**	**df = 15**
B. subcaudals		
	* zweifeli *	* pseudoreginae *
		p = 0.350
* zweifeli *		df = 7
	**p = 0.004**	p = 0.230
* reginae *	**df = 38**	df = 36

## Appendix 4

**Table:** Material used for molecular analysis and GenBank numbers. Key: * sequenced in this study.

Species	Museum voucher	Locality	12S	16S	c-mos
* Erythrolamprus aesculapii *	ROM 47474	Guyana	-	KY986512	KY986488
	IBSP 74046	Brazil	GQ457795	GQ457736	GQ457856
	MNHN 1996.7896	French Guiana	AF158462	AF158531	GQ895814
* Erythrolamprus almadensis *	LSUMZ H-6558	Unknown	–	KY986517	KY986497
	MCP < BRA > 6528	?	JQ598808	JQ598871	JQ598979
* Erythrolamprus atraventer *	IBSP 74342	?	JQ598809	JQ598872	JQ598980
* Erythrolamprus bizona *	LSUMZ H-6360	Costa Rica	–	KY986513	KY986493
* Erythrolamprus breviceps *	MNHN 1996.7879	French Guiana	AF158464	AF158533	–
* Erythrolamprus ceii *	FML 14973	?	JQ598810	JQ598873	JQ598981
* Erythrolamprus cobella *	ROM 28372	Guyana	–	KY986514	KY986489
* Erythrolamprus cursor *	MNHN 1887.0120	Martinique	JX905307	JX905311	–
* Erythrolamprus epinephalus *	LSUMZ H-1547	Peru	–	KY986515	KY986487
	None	Costa Rica	GU018158	GU018176	–
* Erythrolamprus jaegeri *	IBSP 59252	?	GQ457809	GQ457749	GQ457869
* Erythrolamprus juliae *	SBH 194227	Dominica	AF158445	AF158514	–
* Erythrolamprus melanotus *	RML 0266	Tobago	–	KY986510	KY986492
* Erythrolamprus miliaris *	ROM 22837	Guyana	–	KY986511	KY986494
	MZUSP 14137	?	JQ598811	JQ598874	JQ598982
	None	French Guiana	AF158409	AF158480	–
* Erythrolamprus mimus *	LSUMZ H-6398	Honduras	–	KY986508	KY986496
	ICP 1105	Costa Rica	GU018157	GU018175	–
* Erythrolamprus ocellatus *	CAS 245326	Tobago	–	KY986518	KY986490
* Erythrolamprus poecilogyrus *	LSUMZ H-6972	Argentina	–	KY986516	KY986491
	FML 15916	?	JQ598812	JQ598875	–
* Erythrolamprus reginae *	IBSP 72733	?	JQ598813	JQ598876	JQ598983
	MNHN 1996.7846	French Guiana	AF158433	AF158501	
* Erythrolamprus typhlus *	LSUMZ H-17725	Brazil		KY986509	KY986495
	IBSP 70643	?	GQ457811	GQ457751	GQ457871
	None	French Guiana	AF158410	AF158481	–
* Xenodon histricus *	MZUSP 13265	?	–	GQ457753	GQ457873
*Erythrolampruspseudoreginae**	UWIZM.2016.22.45	Tobago	MK287470	MK287477	MK287484
*Erythrolamprusmelanotus**	UWIZM.2011.19.14	Tobago	MK287471	MK287481	–
	UWIZM.2011.25	Trinidad	MK287472	MK287478	MK287485
	UWIZM.2016.22.51	Tobago	MK287473	MK287479	MK287486
	UWIZM.2012.27.26	Tobago	MK287474	MK287480	MK287487
*Erythrolampruszweifeli**	CAS245114	Trinidad	MK287475	MK287482	MK287488
	UWIZM.2014.14	Trinidad	MK287476	MK287483	MK287489

## Appendix 5

**Table:** Table of p-uncorrected distances computed in MEGA7 (under a complete deletion option) of all species shown in Figure 2. The order of specimens from top to bottom follows Figure 2. *Erythrolampruspseudoreginae* is marked in bold type and the genetic distance of its closest species (*E.epinephalus*) as recovered from the phylogenetic tree is shown in bold type and marker with a square.

		1	2	3	4	5	6	7	8	9	10	11	12	13	14	15	16	17	18	19	20	21	22	23	24	25	26	27	28	29	30	31	32	33	34	35	36	37
1	* Xenodon histricus *	–																																				
2	*E.poecilogyrus* (Argentina)	0.053	–																																			
3	*E.ceii* (FML 14973)	0.059	0.006	–																																		
4	*E.poecilogyrus* (FML 15916)	0.059	0.006	0	–																																	
5	*E.miliaris* (French Guiana)	0.059	0.021	0.026	0.026	–																																
6	*E.miliaris* (MZUP 14137)	0.056	0.023	0.029	0.029	0.032	–																															
7	*E.miliaris* (Guiana)	0.056	0.023	0.029	0.029	0.032	0	–																														
8	*E.typhlus* (Brazil)	0.041	0.026	0.032	0.032	0.029	0.026	0.026	–																													
9	*E.reginae* (IBSP 72733)	0.044	0.023	0.029	0.029	0.029	0.023	0.023	0.018	–																												
10	*E.reginae* (French Guiana)	0.053	0.026	0.032	0.032	0.023	0.035	0.035	0.023	0.021	–																											
11	*E.zweifeli* (CAS245114 Trinidad)	0.053	0.032	0.038	0.038	0.035	0.041	0.041	0.029	0.032	0.026	–																										
12	*E.zweifeli* (2014.14 Trinidad)	0.053	0.032	0.038	0.038	0.035	0.041	0.041	0.029	0.032	0.026	0	–																									
13	*E.breviceps* (French Guiana)	0.053	0.009	0.015	0.015	0.018	0.026	0.026	0.023	0.023	0.029	0.029	0.029	–																								
14	*E.epinephalus* (Peru)	0.053	0.009	0.015	0.015	0.012	0.021	0.021	0.018	0.018	0.023	0.029	0.029	0.006	–																							
15	*E.epinephalus* (Costa Rica)	0.085	0.053	0.059	0.059	0.059	0.062	0.062	0.053	0.059	0.07	0.07	0.07	0.05	0.047	–																						
16	***E.pseudoreginae*** (2016.22.45 Tobago)	0.067	0.023	0.029	0.029	0.026	0.035	0.035	0.032	0.032	0.038	0.041	0.041	0.021	0.015	0.047	–																					
17	*E.melanotus* (2016.22.51 Tobago)	0.056	0.023	0.023	0.023	0.032	0.029	0.029	0.032	0.029	0.041	0.038	0.038	0.021	0.021	0.047	0.029	–																				
18	*E.melanotus* (2012.27.26 Tobago)	0.056	0.023	0.023	0.023	0.032	0.029	0.029	0.032	0.029	0.041	0.038	0.038	0.021	0.021	0.047	0.029	0	–																			
19	*E.melanotus* (2011.19.14 Tobago)	0.056	0.023	0.023	0.023	0.032	0.029	0.029	0.032	0.029	0.041	0.038	0.038	0.021	0.021	0.047	0.029	0	0	–																		
20	*E.melanotus* (2011.25 Trinidad)	0.056	0.023	0.023	0.023	0.032	0.029	0.029	0.032	0.029	0.041	0.038	0.038	0.021	0.021	0.047	0.029	0	0	0	–																	
21	*E.melanotus* (Tobago)	0.056	0.023	0.023	0.023	0.032	0.029	0.029	0.032	0.029	0.041	0.038	0.038	0.021	0.021	0.047	0.029	0	0	0	0	–																
22	*E.atraventer* (IBSP 74342)	0.065	0.021	0.026	0.026	0.032	0.035	0.035	0.044	0.038	0.041	0.038	0.038	0.021	0.026	0.07	0.041	0.041	0.041	0.041	0.041	0.041	–															
23	*E.jaegeri* (IBSP 59252)	0.076	0.029	0.035	0.035	0.032	0.032	0.032	0.044	0.038	0.038	0.044	0.044	0.026	0.026	0.067	0.035	0.044	0.044	0.044	0.044	0.044	0.029	–														
24	*E.almadensis* (MCP<BRA>6528)	0.067	0.023	0.029	0.029	0.026	0.029	0.029	0.032	0.038	0.038	0.044	0.044	0.026	0.021	0.05	0.026	0.041	0.041	0.041	0.041	0.041	0.029	0.023	–													
25	*E.almadensis* (LSUMP H–6558)	0.056	0.023	0.029	0.029	0.026	0.029	0.029	0.026	0.032	0.038	0.044	0.044	0.026	0.021	0.05	0.029	0.035	0.035	0.035	0.035	0.035	0.035	0.029	0.018	–												
26	*E.juliae* (Dominica)	0.065	0.021	0.026	0.026	0.023	0.026	0.026	0.029	0.029	0.035	0.041	0.041	0.018	0.012	0.047	0.021	0.026	0.026	0.026	0.026	0.026	0.026	0.026	0.021	0.026	–											
27	*E.cursor* (Martinique)	0.059	0.015	0.021	0.021	0.023	0.032	0.032	0.029	0.029	0.035	0.035	0.035	0.012	0.012	0.05	0.026	0.021	0.021	0.021	0.021	0.021	0.026	0.038	0.032	0.032	0.012	–										
28	*E.typhlus* (IBSN7070643)	0.079	0.044	0.044	0.044	0.047	0.056	0.056	0.053	0.059	0.059	0.053	0.053	0.041	0.041	0.056	0.044	0.041	0.041	0.041	0.041	0.041	0.044	0.05	0.038	0.044	0.035	0.038	–									
29	*E.typhlus* (French Guiana)	0.076	0.035	0.035	0.035	0.038	0.047	0.047	0.044	0.05	0.05	0.05	0.05	0.032	0.032	0.053	0.041	0.035	0.035	0.035	0.035	0.035	0.047	0.047	0.041	0.047	0.026	0.032	0.032	–								
30	*E.cobella* (Guyana)	0.059	0.015	0.021	0.021	0.023	0.032	0.032	0.029	0.029	0.035	0.029	0.029	0.006	0.012	0.047	0.023	0.023	0.023	0.023	0.023	0.023	0.026	0.032	0.032	0.032	0.023	0.018	0.041	0.032	–							
31	*E.aesculapii* (Brazil)	0.062	0.032	0.038	0.038	0.041	0.044	0.044	0.041	0.047	0.041	0.041	0.041	0.029	0.035	0.07	0.038	0.05	0.05	0.05	0.05	0.05	0.038	0.038	0.035	0.038	0.041	0.041	0.059	0.05	0.035	–						
32	*E.ocellatus* (Tobago)	0.056	0.026	0.032	0.032	0.041	0.032	0.032	0.035	0.041	0.041	0.041	0.041	0.029	0.029	0.065	0.032	0.044	0.044	0.044	0.044	0.044	0.038	0.038	0.029	0.038	0.029	0.035	0.053	0.038	0.035	0.012	–					
33	*E.aesculapii* (French Guiana)	0.065	0.032	0.035	0.035	0.041	0.038	0.038	0.041	0.041	0.047	0.053	0.053	0.029	0.029	0.073	0.038	0.05	0.05	0.05	0.05	0.05	0.044	0.044	0.041	0.044	0.041	0.041	0.07	0.056	0.035	0.023	0.023	–				
34	*E.aesculapii* (Guyana)	0.062	0.029	0.032	0.032	0.038	0.035	0.035	0.038	0.038	0.044	0.05	0.05	0.026	0.026	0.07	0.035	0.047	0.047	0.047	0.047	0.047	0.041	0.041	0.038	0.041	0.038	0.038	0.067	0.053	0.032	0.021	0.021	0.003	–			
35	*E.bizona* (Costa Rica)	0.059	0.018	0.023	0.023	0.032	0.035	0.035	0.032	0.038	0.038	0.038	0.038	0.015	0.021	0.065	0.035	0.035	0.035	0.035	0.035	0.035	0.029	0.035	0.035	0.035	0.032	0.026	0.056	0.041	0.021	0.021	0.021	0.026	0.023	–		
36	*E.mimus* (Costa Rica)	0.059	0.018	0.023	0.023	0.032	0.035	0.035	0.032	0.038	0.038	0.038	0.038	0.015	0.021	0.065	0.035	0.035	0.035	0.035	0.035	0.035	0.029	0.035	0.035	0.035	0.032	0.026	0.056	0.041	0.021	0.021	0.021	0.026	0.023	0.006	–	
37	*E.mimus* (Honduras)	0.059	0.018	0.023	0.023	0.032	0.035	0.035	0.032	0.038	0.038	0.038	0.038	0.015	0.021	0.065	0.035	0.035	0.035	0.035	0.035	0.035	0.029	0.035	0.035	0.035	0.032	0.026	0.056	0.041	0.021	0.021	0.021	0.026	0.023	0.006	0	–

## References

[B1] Beard JS (1944) The natural vegetation of the island of Tobago, British West Indies. Ecological Monographs 14: 136–163. 10.2307/1943531

[B2] Brown PA (2013) Bird report Tobago and Trinidad 20^th^ February – 6^th^ March 2013. http://www.surfbirds.com/mb/trips/obago-brown-0413.pdf [accessed May 5, 2015]

[B3] Campbell JA Lamar W (2004) The Venomous Reptiles of the Western Hemisphere, Volumes I and II. Comstock Publishing (Cornell University Press), Ithaca, 1–475 [vol. 1], 477–869 [vol. 2].

[B4] Cope ED (1862) Synopsis of the species of *Holcosus* and *Ameiva*, with diagnoses of new West Indian and South American Colubridae. Proceedings of the Academy of Natural Sciences of Philadelphia. 1: 60–594. https://www.jstor.org/stable/4059427

[B5] Dixon JR (1983a) Systematics of the Latin American snake, *Liophisepinephalus* (Serpentes: Colubridae). In: Rhodin AG Miyata K (Eds) Advances in Herpetology and Evolutionary Biology.Museum of Comparative Zoology, Harvard University, Boston, 132–149.

[B6] Dixon JR (1983b) Systematics of *Liophisreginae* and *L.williamsi* (Serpentes, Colubridae), with a description of a new species. Annals of the Carnegie Museum 52: 113–138. https://biodiversitylibrary.org/page/52426132

[B7] Dowling HG (1951) A proposed standard system of counting ventrals in snakes. British Journal of Herpetology 1: 97–99. 10.2307/1437542

[B8] Escalona MD (2017) Range extension for *Erythrolamprusepinephalusbimaculatus* (Cope, 1899) and *E.e.opisthotaenius* (Boulenger, 1908) in Venezuela (Serpentes: Colubridae). Herpetology Notes. 10: 511–5. https://biotaxa.org/hn/article/view/30217/29527

[B9] Esqueda LF Natera-Mumaw M La Marca E (2009) First record of salamander predation by a Liophis (Wagler, 1830) snake in the Venezuelan. Acta Herpetologica 4: 171–175.

[B10] Fitzinger L (1843) Systema Reptilium. Fasciculus primus, Amblyglossae, Braumüller et Seidel, Vindobonae. 10.5962/bhl.title.4694

[B11] Gouy M Guindon S Gascuel O (2010) SeaView version 4. A multiplatform graphical user interface for sequence alignment and phylogenetic tree building. Molecular Biology and Evolution 27: 221–224. 10.1093/molbev/msp259 19854763

[B12] Grazziotin FG Zaher H Murphy RW Scrocchi G Benavides MA Zhang YP Bonatto SL (2012) Molecular phylogeny of the new world Dipsadidae (Serpentes: Colubroidea): a reappraisal. Cladistics 28: 437–459. 10.1111/j.1096-0031.2012.00393.x 34836446

[B13] Hardy JD (1982) Biogeography of Tobago, West Indies, with special reference to amphibians and reptiles, a review. Bulletin of the Maryland Herpetological Society 18: 37–142.

[B14] Hedges SB Duellman WE Heinicke MP (2008) New World direct-developing frogs (Anura: Terrarana): molecular phylogeny, classification, biogeography, and conservation. Zootaxa (1737): 1–182. http://www.mapress.com/j/zt/article/view/4661

[B15] Helmer EH Ruzycki TS Benner J Voggesser SM Scobie BP Park C Fanning DW Ramnarine S (2012) Detailed maps of tropical forest types are within reach: Forest tree communities for Trinidad and Tobago mapped with multiseason Landsat and multiseason fine-resolution imagery. Forest Ecology and Management 279: 147–166. 10.1016/j.foreco.2012.05.016

[B16] Hodson EE Lehtinen RM (2017) Diverse Evidence for the Decline of an Adaptation in a Coral Snake Mimic. Evolutionary Biology 44: 401–10. 10.1007/s11692-017-9418-7

[B17] Huelsenbeck JP Ronquist F (2001) MrBayes: Bayesian inference of phylogeny. Bioinformatics 17: 754–755. 10.1093/bioinformatics/17.8.754 11524383

[B18] Jan G (1863) Enumerazione sistematica degli ofidi appartenential gruppo Coronellidae. Archivio per la Zoologia, l’Anatomia, e la Fisiologia 2: 213–330.

[B19] Jowers MJ Caut S Garcia-Mudarra JL Alaasad S Ineich I (2013) Molecular phylogenetics of the possibly extinct Martinique ground snake. Herpetologica 69: 227–236. 10.1655/HERPETOLOGICA-D-12-00085

[B20] Jowers MJ Lehtinen RM Downie RJ Georgiadis AP Murphy JC (2015) Molecular phylogenetics of the glass frog *Hyalinobatrachiumorientale* (Anura: Centrolenidae): evidence for Pliocene connections between mainland Venezuela and the island of Tobago. Mitochondrial DNA (2014): 1–6. 10.3109/19401736.2014.880888 24491102

[B21] Katoh K Misawa K Kuma K Miyata T (2002) MAFFT: a novel method for rapid multiple sequence alignment based on fast Fourier transform. Nucleic Acids Research 30: 3059–66. 10.1093/nar/gkf436 PMC135756 12136088

[B22] Kocher TD Thomas WK Meyer A Edwards SV Pääbo S Villablanca FX Wilson AC (1989) Dynamics of mitochondrial DNA evolution in animals: amplification and sequencing with conserved primers. Proceedings of the National Academy of Sciences 86: 6196−200. 10.1073/pnas.86.16.6196 PMC297804 2762322

[B23] Kumar S Stecher G Tamura K (2016) Molecular evolutionary genetics analysis version 7.0. for bigger datasets. Molecular Biology and Evolution 33(7): 1870–1874. 10.1093/molbev/msw054 27004904 PMC8210823

[B24] Lanfear R Calcott B Simon YW Guindon S (2012) PartitionFinder: Combined selection of partitioning schemes and substitution models for phylogenetic analyses. Molecular Phylogenetics and Evolution 28: 1695–1701. 10.1093/molbev/mss020 22319168

[B25] Lehtinen RM Wojtowicz EA Hailey A (2011) Male vocalizations, female discrimination, and molecular phylogeny: multiple perspectives on the taxonomic status of a critically endangered Caribbean frog. Journal of Zoology 283: 117–125. 10.1111/j.1469-7998.2010.00752.x

[B26] Linnaeus C (1758) *Systema naturæ per regna tria naturæ*, *secundum classes*, *ordines*, *genera*, *species*, *cum characteribus*, *differentiis*, *synonymis*, *locis* Tomus I. Editio decimal, reformata. Laurentii Salvii, Holmiæ (10^th^ edn). 10.5962/bhl.title.542

[B27] Manzanilla J La Marca E García‐París M (2009) Phylogenetic patterns of diversification in a clade of Neotropical frogs (Anura: Aromobatidae: *Mannophryne*). Biological Journal of the Linnean Society 97: 185–199. 10.1111/j.1095-8312.2009.01074.x

[B28] Michaud EJ Dixon JR (1989) Prey items of 20 species of the neotropical colubrid snake genus *Liophis*. Herpetological Review 20: 39−41.

[B29] Miller MA Pfeiffer W Schwartz T (2010) Creating the CIPRES Science Gateway for inference of large phylogenetic trees. Proceedings of the Gateway Computing Environments Workshop (GCE), 14 Nov. 2010, New Orleans, LA, 1–8. 10.1145/2016741.2016785

[B30] Murphy JC (1997) Amphibians and reptiles of Trinidad and Tobago. Krieger Publishing, Malabar, Florida, 245 pp 10.2307/1447462

[B31] Murphy JC Downie JR Smith JM Livingstone SR Mohammed RS Lehtinen RM Eyre M Sewlal J Noriega N Caspar GS Anton T Rutherford MG Braswell AL Jowers MJ (2018) A field guide to the amphibians and reptiles of Trinidad and Tobago. Trinidad and Tobago Field Naturalists’ Club, Port of Spain, 336 pp.

[B32] Myers CW (2011) A new genus and new tribe for *Enicognathusmelanauchen* Jan, 1863, a neglected South American snake (Colubridae: Xenodontinae), with taxonomic notes on some Dipsadinae. American Museum Novitates 715: 1–33. http://hdl.handle.net/2246/6115

[B33] Palumbi S (1996) Nucleic acids II: The polymerase chain reaction. In: Hillis DM Moritz C Mable BK (Eds) Molecular Systematics.Sinauer, Sunderland, MA, 205–248.

[B34] Rambaut A Suchard MA Xie D Drummond AJ (2014) Tracer v1.6. http://beast.bio.edu.ac.uk/Tracer

[B35] Rivas GA Molina CR Ugueto GN Barros TR Barrio-Amorós CL Kok PJP (2012) Reptiles of Venezuela: an updated and commented checklist. Zootaxa 3211: 1–64.

[B36] Roze JA (1958a) Resultados zoologicos de la expedicion de la Universidad Central de Venezuela a la region del Auyante’pui en la Guyana Venezolana, Abril de 1956. 5. Los reptiles del Auyantepui, Venezuela, basandose en las colecciones de las expediciones de Phelps-Ta Acta Biologica Venezuelica 2: 243–270.

[B37] Roze JA (1958b) Los reptiles del Chimantá Tepui (Estado Bolívar, Venezuela) colectados por la expedición botánica del Chicago Natural History Museum. Acta Biologica Venezuelica 2: 299–314.

[B38] Roze JA (1959) Taxonomic notes on a collection of Venezuelan reptiles in the American Museum of Natural History. American Museum Novitates (1934): 1−14. http://hdl.handle.net/2246/4351

[B39] Roze JA (1964) The snakes of the *Leimadophis-Urotheca-Liophis* complex from Parque Nacional Henri Pittier (Rancho Grande), Venezuela, with a description of a new genus and species (Reptilia, Colubridae). Senckenbergiana Biologica 45: 533−542.

[B40] Roze JA (1966) La Taxonomia y Zoogeographia de los Ofdios de Venezuela. Ediciones de la Biblioteca 28, Caracas, 357 pp.

[B41] Saint KM Austin CC Donnellan SC Hutchinson MN (1998) C-mos, a nuclear marker useful for squamate phylogenetic analysis. Molecular Phylogenetics and Evolution 10: 259–263. 10.1006/mpev.1998.0515 9878236

[B42] Savage JM (2002) The Amphibians and Reptiles of Costa Rica, a herpetofauna between two continents between two seas. The University of Chicago Press, Chicago, 943 pp.

[B43] Shaw G (1802) General zoology or systematic natural history. 3, Pt 1. Thomas Davison, London. 10.5962/bhl.title.1593

[B44] Silva JL Valdez J (1989) Ritmo diario de actividad y periodo de ecolosión de algunos ofidios del Norte de Venezuela. Acta Biológica Venezuelica 12: 88–97. https://biblat.unam.mx/fr/revista/acta-biologica-venezuelica/15

[B45] Silva JL Valdez J Ojasti O (1985) Algunos aspectos de una comunidad de ofidios del Norte de Venezuela. Biotropica 17: 112–125. 10.2307/2388503

[B46] Silvestro D Michalak I (2010) A user-friendly graphical front-end for phylogenetic analyses using RAxML (Stamatakis, 2006). Organisms Diversity and Evolution 12: 335–337. 10.1007/s13127-011-0056-0

[B47] Sisson VB Avé Lallemant HG Ostos M Blythe AE Snee LW Copeland P Wright JE Donelick RA Guth LR (2005) Overview of radiometric ages in three allochthonous belts of northern Venezuela: Old ones, new ones, and their impact on regional geology, in Avé Lallemant, HG, Sisson VB (Eds) Caribbean–South American plate interactions, Venezuela: Geological Society of America Special Paper 394: 91–117. 10.1130/0-8137-2394-9.91

[B48] Uetz P Freed P Hošek J [Eds] (2018) The Reptile Database. http://www.reptile-database.org [accessed June 5, 2018]

[B49] Vidal N Dewynter M Gower DJ (2010) Dissecting the major American snake radiation: a molecular phylogeny of the Dipsadidae Bonaparte (Serpentes, Caenophidia). Comptes Rendus Biologies 333: 48−55. 10.1016/j.crvi.2009.11.003 20176336

[B50] Wallach V Williams KL Boundy J (2014) Snakes of the World: A catalogue of living and extinct species. CRC Press, Boca Ratan, 1209 pp 10.1201/b16901

[B51] Zaher H Grazziotin FG Cadle JE Murpht RW Moura-Leite JCD Bonatto SL (2009) Molecular phylogeny of advanced snakes (Serpentes, Caenophidia) with an emphasis on South American Xenodontines: a revised classification and descriptions of new taxa. Papéis Avulsos de Zoologi 49: 115–153. 10.1590/S0031-10492009001100001

